# TRANSITIONAL PALLIATIVE CARE FOR RURAL FAMILY CAREGIVERS USING VIDEO TECHNOLOGY: RECRUITMENT, METHODS, AND FINDINGS

**DOI:** 10.1093/geroni/igae098.2110

**Published:** 2024-12-31

**Authors:** Joan Griffin

**Affiliations:** Chair

## Abstract

People with severe, life-limiting illnesses face serious risks for adverse events and symptom escalation when transitioning care from hospital to home. When new approaches to care, such as palliative care, are introduced before discharge, these risks increase further. Family caregivers (FCG) often help manage care during these transitions and ensure care recipient safety and quality of care after discharge. These new or added responsibilities can strain FCG health and well-being, thus compromising FCG and care recipient health. This is especially true for FCGs in rural and underserved areas who typically have fewer resources and limited access to care. This symposium presents lessons learned, methodological challenges, and findings from a FCG study aimed at addressing transitional care and palliative care on FCGs health and well-being. The intervention tested a model of care to address unmet needs of FCGs caring for seriously ill and elderly patients in rural areas through teaching, guidance, and counseling via video visits. Preliminary findings show this intervention improves FCG outcomes and is cost-effective. The first presentation provides an overview of the trial (Griffin). The second presentation describes challenges with recruiting and differences between recruiting FCG participants in-person and remotely (Gustafson). The third presentation includes findings on internet connectivity when conducting a virtual clinical trial with FCGs living in rural and underserved areas (Holland). The fourth presentation describes study findings on FCG and care recipient health and well-being (Griffin). The fifth presentation includes a discussion of the differences in caregiving and utilization costs associated with the intervention (Kaufman).

